# Retrospective analysis of management of ingested foreign bodies and food impactions in emergency endoscopic setting in adults

**DOI:** 10.1186/s12873-016-0104-3

**Published:** 2016-11-04

**Authors:** Girolamo Geraci, Carmelo Sciume’, Giovanni Di Carlo, Antonino Picciurro, Giuseppe Modica

**Affiliations:** Operative Unit of General and Thoracic Surgery, University of Palermo, Palermo, Italy

**Keywords:** Upper endoscopy, Foreign body, Food bolus impaction, Endoscopic management

## Abstract

**Background:**

Ingestion of foreign bodies and food impaction represent the second most common endoscopic emergency after bleeding. The aim of this paper is to report the management and the outcomes in 67 patients admitted for suspected ingestion of foreign body between December 2012 and December 2014.

**Methods:**

This retrospective study was conducted at Palermo University Hospitals, Italy, over a 2-year period. We reviewed patients’ database (age, sex, type of foreign body and its anatomical location, treatments, and outcomes as complications, success rates, and mortalities).

**Results:**

Foreign bodies were found in all of our 67 patients. Almost all were found in the stomach and lower esophagus (77 %). The types of foreign body were very different, but they were chiefly meat boluses, fishbones or cartilages, button battery and dental prostheses. In all patients it was possible to endoscopically remove the foreign body. Complications related to the endoscopic procedure were unfrequent (about 7 %) and have been treated conservatively. 5.9 % of patients had previous esophageal or laryngeal surgery, and 8.9 % had an underlying esophageal disease, such as a narrowing, dismotility or achalasia.

**Conclusion:**

Our experience with foreign bodies and food impaction emphasizes the importance of endoscopic approach and removal, simple and secure when performed by experienced hands and under conscious sedation in most cases.

High success rates, lower incidence of minor complications, reduction of the need of surgery and reduced hospitalization time are the strengths of the endoscopic approach.

## Background

Foreign-object ingestion and food-bolus impaction are common occurrence in the emergency endoscopy. They represent a significant clinical problem, causing a high degree of financial burden, morbidity and mortality, and pose diagnostic and sometimes therapeutic challenges. In adults, foreign-object ingestion or insertion occurs more commonly among those with psychiatric disorders or mental retardation, as well as food impactions or impairment occurs more commonly in subject with previous upper laryngeal or gastrointestinal surgery and in case of altered esophageal motility [1–3]; moreover, their management depend on a number of factors, such as anatomic location, shape and size of the foreign body, and duration of impaction [1].

The aim of our retrospective study is to report our experience and outcome in the management of 67 consecutive cases of ingestion of foreign bodies or food impactions in a University Hospital with emergency endoscopy setting.

## Methods

### Data collection

Sixty-seven consecutive patients (27 % of 241 consecutive admissions, 50 male and 17 female, mean age 47 years, range 19–62 years, median age 53 years) with a recent history of foreign body ingestion or food bolus impaction were admitted over a 2-year period between December 2012 and December 2014, from the Emergency Room to the Emergency Digestive Endoscopy Service of University Hospital “Paolo Giaccone” in Palermo.

### Clinical practice

A previous clinical history of foreign body ingestion was present in six cases (9 %).

Forty-three patients referred no comorbidities related to altered transit; whereas, schizophrenia was reported in 1 patient, esophageal narrowing in 2, esophageal dismotility in 2, achalasia in 2, previous laryngeal surgery (tracheostomy tube) in 2, previous esophageal surgery in 1 and drug addiction in 14 patients.

Nineteen patients (28.79 %) were asymptomatic, while 43 patients (65.15 %) referred dysphagia, 12 (18.18 %) nausea, 9 (13.6 %) salivation, 7 (10.6 %) drooling, 6 (9 %) vomiting, 1 (1.5 %) gastric outlet obstruction and 1 (1.5 %) sense of lump behind the sternum.

The clinical suspect was supported by radiographic findings in 48 cases (72 %), performed within 5 h form diagnosis (plain radiography of the abdomen in 34 cases = 70 %, plain radiography of the neck and chest in 11 cases = 23 %, CT in 9 = 18 %, and abdominal ultrasound in 3 cases = 6 %) and always before endoscopy, also to rule out the suspicion of perforation.

In 17 cases (25.3 %) the ingestion was voluntary.

All patients were asked to give their informed consent and no one refused (the patients with psychiatric disturbance received consent from legal guardian).

All patients received emergency upper endoscopy within 6 h of ingestion and were followed until elimination or removal of the foreign objects.

The endoscopies were all performed by two endoscopic surgeon, in collaboration with a specialized nurse and an anesthesiologist.

The procedure was performed under local pharyngeal anesthesia (Lidocaine chloridrate spray 10 % 10 gr/100 ml, Molteni Farmaceutica, Firenze, Italy) and a combination of midazolam and fentanyl, on escalating dosing according to the needs for conscious sedation in 51 cases (75 %); 16 patients (25 %) refused sedation.

The patients with psychiatric disturbance underwent the same type of analgosedation.

The procedures were conducted under heart rate, oximetry and and blood pressure monitoring and supplemental oxygen was given through nasal mask during the entire procedure.

We used flexible endoscopes (GIF-Q145, GIF-Q165 and GIF-Q180; Olympus Optical Co, Ltd, Hamburg, Germany) and accessories used to remove the foreign bodies included snares, forceps and retrieval basket.

Demographic and endoscopic data, including age, sex, referral sources of patients, types, number, and dimension and location of foreign bodies or food bolus impacted, associated upper-GI disease, endoscopic methods and accessory devices for removal of foreign bodies or food bolus were retrospectively collected and analyzed (Table 1). The patients were observed until hospital discharge.

**Table 1:**
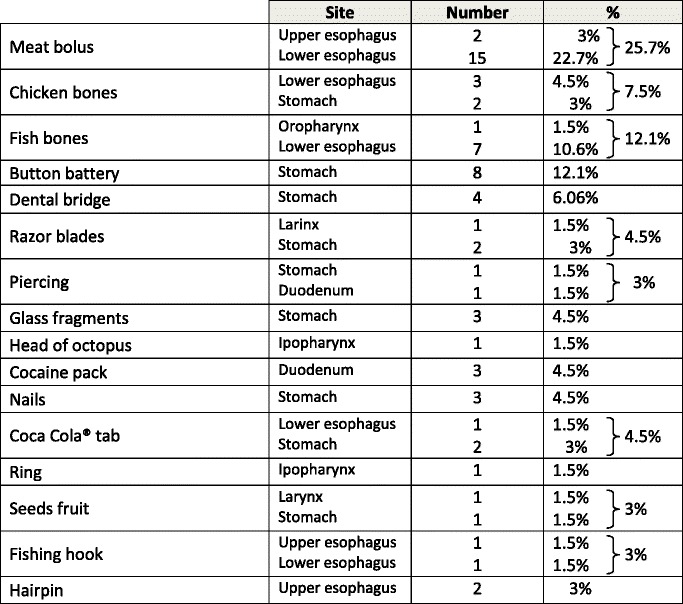
Type and localization of foreign bodies (also contemporary)

### Statistics

All data correspond to a normal Gaussian distribution, according to tests performed before and after data collections (the value of mean corresponds to the value of the median, and asymmetry is 0.51, between the value of −2 to +2).

GG and GCD reviewed and collected all patients’ files from intranet hospital database with full notations on the following data: age, sex, type of foreign body, its anatomical location, treatments, and outcomes (complications, success rates, and mortalities); GG and CS reviewed the charts and a third blind observer (statistical doctor) confirmed correct data extraction and entry.

Our study received approval by the Institutional Ethic Committee of Faculty of Medicine.

## Results

The foreign bodies have been identified through upper endoscopy in all 67 patients referred to us because of suspected foreign-body ingestion.

The types of foreign bodies found in the upper-GI tract varied greatly, including in order of frequency, food-bolus impactions (17 patients), fish bones (8 patients), button battery (8 patients), chicken bones (5 patients), dental bridge (4 patients), razor blades (3 patients), glass fragments (3 patients), cocaine packs (3 patients), nails (3 patients), coca cola tabs (3 patients), piercing (2 patients), seeds fruit (2 patients), fishing hook (2 patients), hairpin (2 patients), rings (1 patient) and head of octopus (1 patient).

Fish and chicken bones and dental prostheses were the most common foreign bodies elderly people.

The foreign bodies were located more frequently in the lower esophagus (27 patients = 40.5 %) and in the stomach (27 patients = 40.5 %), followed by upper esophagus (4 patients = 6.06 %), duodenum (4 patients = 6.06 %), larynx (2 patients = 3.04 %), ipopharynx (2 patients = 3.04 %) and oropharynx (1 patient = 1.5 %).

The most common foreign bodies in the pharynx and the operated pharynx (also with tracheostomy tube) were bones and food-bolus impactions, respectively. Fish bones, food bolus, and dental prostheses were the most common foreign bodies in the esophagus, whereas button batteries and dental bridge were frequently located in the stomach. In the duodenum, the foreign bodies that had passed through the stomach were small and smoothly shaped objects, such as piercing and little cocaine pack.

### Method and technique of upper endoscopy

The endoscopic methods varied according to the types of the foreign bodies and the more frequently used accessory devices were retrieval Dormia basket (57 %) followed by rat-tooth foreign bodies forceps (24 %) and snare (19 %).

Dormia basket was the preferred method to retrieve button batteries (100 %), piercing (100 %), glass fragments (100 %).

Pulling with a rat-tooth forceps was the most effective method to extract the chicken and fish bones (100 %). Snares were most frequently used in dealing with the ingested dental prostheses. Large or small food bolus, after fragmentation, were gently pushed into the stomach or intestine by gentle pressure with the endoscope on the center of the bolus.

A latex protector hood or an overtube was used to protect the esophageal mucosa during procedure.

In summary, we performed endoscopic extraction in 49 cases (73 %) and dislodgement in 18 (27 %).

There was no mortality associated with the endoscopic procedures of removing foreign bodies in our center over the past 2 years. The complications of the endoscopic procedure included mucosal laceration (5 cases = 7.5 %) and fever ≤38 °C (4 cases = 6 %).

Mucosal laceration were immediately treated by endoscopy clipping without further morbidity, and the patients with fever were recovered after administration of broad spectrum antibiotics for 2 days.

## Discussion

Foreign-object ingestion and food-bolus impaction are common occurrence all over the world: 80 to 90 % of foreign bodies ingested have been reported to pass harmlessly and spontaneously through the GI tract, although approximately 1500 deaths per year have been attributed to foreign-body ingestions in the USA [1, 4]; in Italy the overall incidence is about 450 new cases/year (60 % in children <4 years) [2, 3].

Nevertheless, mortality rates have been extremely low: a compilation of multiple studies including 2 large series report no deaths in 852 adults and 1 death in 2206 children [5].

The mortality associated is really unknown [5], nevertheless it is very low because of the high rate of early and resolutive endoscopic removal (63-76 %) [5], as well as the rate of spontaneous passage, without intervention or any damage of gastrointestinal tract, was related to the size and the type of foreign bodies (it has been suggested by the ASGE that only 10 to 20 % of foreign bodies may need to be removed endoscopically) [5, 6].

Since the first report in 1972 on the endoscopic removal of a foreign body [7] there has been an increasing request of endoscopic maneuvers, because of avoidance of surgical removal for most patients reduced cost; besides, endoscopic removal is characterized by technical facility, excellent visualization, simultaneous diagnosis of other diseases, and a low rate of morbidity [5].

Direct radiographs may identify most radiopaque foreign bodies but not food bolus impaction; in addition, fish or chicken bones, wood, plastic, most glass, and thin metal objects are not readily seen [5].

Swelling, erythema, tenderness, or crepitus in the neck region may be present with oropharyngeal or proximal esophageal perforation and the abdomen should be examined for evidence of peritonitis or signs of obstruction; these conditions will require surgical intervention and consultation should not be delayed for endoscopy [8].

The increased salivation, as in our cases, is a sign of proximal esophageal obstruction (9 cases = 13.6 %) [7], and may lead to early endoscopy, while dislodgment of the fleshy meat bolus from the esophagus was achieved by gentle pressure with the endoscope on the center of the bolus [9].

Nevertheless, in clinical endoscopic practice, if the risk of esophageal perforation and bleeding is high, as in those cases with sharpened or pointed foreign bodies deeply fixed into the wall, it is better to avoid any endoscopic attempts and to resort to surgery [6].

The open safety pin always represented a major problem: if a safety pin is in the esophagus with the open end proximal, it is best managed with the flexible endoscope by pushing the pin into the stomach, turning it, and then grasping the hinged end and pulling it out first. The use of overtube makes easier the extraction. The ingested razor single-edge blade is also a traumatic experience for both the patient and the endoscopist, but it can be managed with the flexible endoscope and overtube, especially if it has reached the stomach. One also can use a rubber hood or a self made piece of rubber glove (finger glove) on the tip of the endoscope to protect the esophagus from sharp or pointed foreign bodies. Once a razor blade has negotiated the stomach, surprisingly, it will usually pass through the lower gastrointestinal tract without difficulty [10].

Sites of trapped foreign bodies or food bolus may be related to at least three factors: anatomical (narrowest areas as upper esophagus, were a common site, especially in the elderly with neurological deficits); pathological (acquired strictures); and the nature of foreign body: sharp pins were mostly seen piercing the antrum. This, in turn, determined the tools to be used in removal: lodged foreign bodies or food impaction were grasped by forceps while in the stomach, it was easy to use the snare or to open and close the basket [9].

Most ingested foreign bodies (80–90 %) pass through the GI tract spontaneously, 10 to 20 % need endoscopic intervention, and only 1 % or fewer may require surgery [4, 11]: objects larger than 2 cm in diameter may lodge at the pylorus, whereas objects longer than 6 cm may become entrapped either at the pylorus or at the duodenal C-curve, between the first, second and third part of the duodenum, and rarely pass beyond that point [3]; besides, a large foreign body, occluding the visceral lumen, may lead to severe symptoms and even death, whereas a small foreign body should be asymptomatic, apart from a recent history of foreign body ingestion [4, 9].

The size, shape, and classification of the ingested material, the anatomic location in which the object is localized and, finally, the endoscopist’s skill influence the management and practice at grasping a identical or similar object with the available instruments outside the patient may be beneficial [9, 12].

Overtube offers airway protection during retrieval, allows for multiple passes of the endoscope during removal of multiple foreign bodies or a food impaction, and may protect the esophageal mucosa from lacerations during retrieval of sharp objects [4, 13].

If the foreign body becomes impacted in the esophagus, it may cause serious sequelae such as esophagitis, mucosal ulceration and hemorrhage, obstruction, perforation, or, rarely, death [11].

Significant factors that might predispose patients to complications include delayed presentation (≤24 h after the onset of symptoms), presence of a sharp foreign body, mental illness, wearing dentures, and multiple objects. A high index of suspicion, judicious judgment, and early endoscopic intervention within 24 h after ingestion are associated with favorable outcomes [11].

Likewise, we found that about 58 % of our patients had an impaction in the esophagus, and for this reasons, physicians should examine this organ more carefully both to identify the foreign body and to assess the condition of the mucosa [9].

Because of the ingestion is often in proximity to a meal, the risk of aspiration should be assessed, and the ventilation and the airway should be secured, also with tracheal intubation, if necessary [5].

From what was previously written, it is clear that experienced endoscopists and well-equipped theaters are required to perform these maneuvers [5].

In our experience, according to international indications, the endoscopic procedure was performed in most of the patients within 6 h, because the foreign bodies had not passed through the upper-GI tract.

## Conclusions

Ingestion of foreign bodies is a worldwide common clinic problem and an endoscopic conservative approach should be shortly always performed for its excellent success rate (≥90 %) and lower failure rate or minor complication.

Our experience with endoscopic foreign body removal emphasizes its importance and ease when performed by experienced hands with tailored approach, at well-equipped endoscopy units, and under conscious sedation in most cases, with high success rates and minor complications.

However, majority of cases can be successfully managed conservatively.

In our experience, in accord to international literature, surgery is required only in selected patients with high risk of esophageal perforation or after removal failure of sharpened objects.
